# Drinking from the Holy Grail—Does a Perfect Triage System Exist? And Where to Look for It?

**DOI:** 10.3390/jpm14060590

**Published:** 2024-05-31

**Authors:** Anna Ingielewicz, Piotr Rychlik, Mariusz Sieminski

**Affiliations:** 1 Department of Emergency Medicine, Faculty of Health Science, Medical University of Gdansk, Mariana Smoluchowskiego Street 17, 80-214 Gdansk, Poland; sieminski@gumed.edu.pl; 2 Emergency Department, Copernicus Hospital, Nowe Ogrody Street 1-6, 80-203 Gdansk, Poland

**Keywords:** triage system comparison, in-hospital triage, medical segregation, emergency severity index, Manchester triage system

## Abstract

The Emergency Department (ED) is a facility meant to treat patients in need of medical assistance. The choice of triage system hugely impactsed the organization of any given ED and it is important to analyze them for their effectiveness. The goal of this review is to briefly describe selected triage systems in an attempt to find the perfect one. Papers published in PubMed from 1990 to 2022 were reviewed. The following terms were used for comparison: “ED” and “triage system”. The papers contained data on the design and function of the triage system, its validation, and its performance. After studies comparing the distinct means of patient selection were reviewed, they were meant to be classified as either flawed or non-ideal. The validity of all the comparable segregation systems was similar. A possible solution would be to search for a new, measurable parameter for a more accurate risk estimation, which could be a game changer in terms of triage assessment. The dynamic development of artificial intelligence (AI) technologies has recently been observed. The authors of this study believe that the future segregation system should be a combination of the experience and intuition of trained healthcare professionals and modern technology (artificial intelligence).

## 1. Introduction

The Emergency Department (ED) is dedicated to patients requiring urgent medical assistance. Globally, EDs are faced with an inflow of patients who outnumber their capacity. Moreover, a consequent year-to-year increase in the number of patients visiting EDs has been observed globally [1,2]. ED patients are not only growing in number but are also growing older. Approximately 30% of the visitors to EDs are above 65 years old with medical consequences of advanced age, such as the fragility syndrome, multiple comorbidities, and a higher risk of life-threatening conditions [3,4]. This overcrowding in EDs results in an increase in burden on the medical staff, need for resources, boarding time, and time to treatment [5,6]. A crucial organizational need in EDs is the proper allocation of resources to provide medical services to patients on time, adjusted to the urgency of their condition. One of the tools for solving this challenge is the medical triage.

The in-hospital medical triage is aimed at the prioritization of patients to determine the time during which physicians should assess them. It facilitates the flow of patients within the ED [1,3,5,6]. The emergency room triage is usually performed by a trained nurse or paramedic who assesses patients’ signs and symptoms as well as vital signs. Additionally, the in-hospital triage aims to determine the amount and type of resources required for patients as well as allocation of these resources to provide care in time to patients according to their severity [1,3,6]. Experiments were conducted to assess the effect of replacing nurse-performed triage systems with AI-run triage systems [7,8], or by the patients themselves, but results have not been clear [9].

On the other hand, prehospital triage has also been postulated and assessed in scientific projects [10,11,12,13], as well as using machine learning models to optimize care in emergency services e.g., chest pain, trauma brain injuries, or ophthalmic problems [14,15,16]. The results of the mentioned solutions have increasingly strong evidence based in science, but further studies are required [17].

The purpose of this study is to review the literature on the topic of triage to find an answer to the question of whether an ideal segregation system currently exists, and if not, where to look for it. In this paper, we briefly characterize the selected triage systems in an attempt to describe an ideal triage system and review the results of comparisons among the studies on the various triage systems. The authors know that other systems of medical triage with proven usefulness exist; however, the choice of systems compared in this article resulted from the fact that they are a reference point for most of the world’s publications [18,19,20,21]. To select sources for this review, PubMed search was performed from 1990 to April 2024. The following terms were used for comparison: “ED”, “triage system”, “triage system comparison”, and “artificial intelligence”. Publications available in English were selected for analysis. The first search identified 700 publications. After reading the abstracts, 126 were singled out and were then read in full. After a comprehensive study of the publications, 66 articles were selected for final analysis.

The papers contain data about the following:

(a) The design and function of a triage system
(b) Its validation
(c) Its performance
(d) The comparison between the two triage systems

The use of machine-learning methods in the triage process was first analyzed, and the final reference list was created based on the relevance to the topic of the review.

Many triage systems have been created and used in hospitals worldwide, such as the Manchester Triage System (MTS) [5,22], Emergency Severity Index (ESI) [23,24,25], Canadian Triage and Acuity Scale (CTAS) [26], and Australian Triage Scale (ATS) [27]. Triage systems based on a five-grade scale are the most common, with scientifically proven superiority to the three-grade solutions [28,29,30].

The patient’s urgency is coded by the color or the digit, descending from the most urgent cases (e.g., red—1; orange—2; Yellow—3; green—4. Blue—5). While the two top grades represent a minority of patients and are served immediately in most cases, the most common are the three lowest categories. This is a confusion-generating disadvantage of the triage systems, because of which the ED staff face a high number of patients with similar priorities, among whom patients still require urgent help [4,31]. The latter is another problem of the triage system in terms of sensitivity and specificity.

The triage system used plays a pivotal role in the organization of the ED’s functions. Therefore, the criteria which is useful in the assessment of triage systems, and for comparative studies focused on the analysis of the specificity and sensitivity of triage systems, is required in the context of the effective screening of patients at risk of death, cardiac arrest, immediate intervention (e.g., the percutaneous coronary intervention—PCI), and hospitalization in the Intensive Care Unit (ICU). This article deals with issues rarely discussed on this topic, such as the ease of use of the triage system, the time needed to learn it, and the diversity of triage use in different groups of patients. The authors did not find any papers addressing all the issues discussed in this article.

## 2. Ideal Triage System

**Figure 1:**
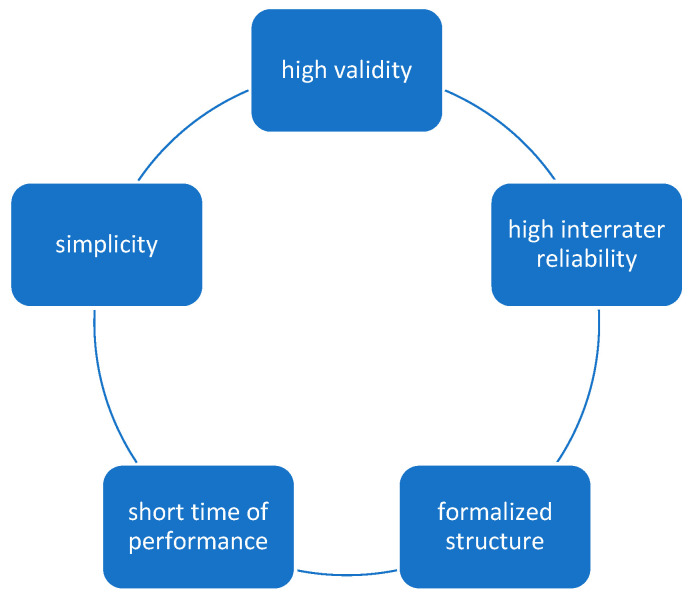
Features of an ideal in-hospital triage system.

As mentioned above, triage systems presently used worldwide are five-degree scales in most cases. The most common systems that were created in the last decade of the previous century have undergone constant optimization until now. Since their creation, their authors have been making efforts to analyze whether the triage systems meet the needs of patients and ED staff, and the results of studies in that area have been used to improve the systems. This has been carried out to design a system characterized as follows (Figure 1):

High validity is defined as the ability of a system to prioritize patients concordantly with their actual conditions. The difficulty achieving this goal lies in the lack of an ideal benchmark (a “gold standard”) to which the priority established during triage could have been compared.

High interrater reliability means the repeatability of the results regardless of the person performing the triage. It is expressed by Cohen’s kappa coefficient, taking values from 0.0 to 1.0—the closer to 1.0, the larger the agreement between the raters’ results.

Simplicity is understood as the ease of proceeding with the triage. This can be measured using users’ satisfaction. This parameter may impact the kappa coefficient described above and is related to the time required for training during triage.

Short time of performance means the time taken for diagnosis and treatment—the actual aims behind visiting the ED. The authors of the systems of triage focus on time, for example, assuming that it is supposed to last no longer than two minutes.

Formalized structure is important because using the system is described by an algorithm of triage that influences the kappa coefficient and improves staff training.

Combining all the above elements presently seems impossible therefore, systems that compromise these expectations are used at the ED.

## 3. Most Common Triage Systems

To discuss all medical segregation systems in the world is impossible, and a few of the most common systems will be highlighted here. Because of the higher reliability proven in many studies, only five-step systems [2] will be presented in the alphabetical order:

I. ATS (Australian Triage Scale)

The system was introduced in the Emergency Departments in Australia in 1994 and was closely related to the patient’s waiting time for a medical examination:

- 0 min. for priority 1 (red)
- Up to 10 min for priority 2 (orange)
- Up to 30 min for priority 3 (green)
- Up to 60 min for priority 4 (blue)
- Up to 120 min. for priority 5 (white) [27].

At the same time, it is noted that the system’s task is to assess the urgency of providing help, not necessarily the severity of the patient’s condition.

The nurse has at his disposal a table presenting five priorities, with the discriminators listed next to them, which are a description of clinical situations, e.g., suspected sepsis (hemodynamically unstable), testicular torsion, severe pain.

The nurse conducting the assessment determined the reason for the report. The triage process is a two-stage process, it begins with an assessment according to the ABCD scheme, with the task of establishing at the outset whether the patient is unstable and requires immediate care. If not, a more complete assessment is made, based on discriminators.

The system includes additional lists of discriminators for patients with mental disorders and for children, vital parameters are checked although they do not directly influence the result of the scale.

II. CTAS (Canadian Triage and Acuity Scale)

The system was developed in the 1990s in Canada, based on the ATS system. It is related to the specific waiting times for the first medical evaluation.

- For priority 1 (blue), assistance must be provided immediately,
- Priority 2 (red) can wait up to 15 min,
- Priority 3 (yellow) waiting time up to 30 min,
- Priority 4 (green) waiting time up to 60 min,
- Priority 5 (white) waiting time up to 120 min [26].

This system also includes the obligation to conduct re-triage in intervals, depending on the priority, which coincides with the patient’s time of acceptable expectation.

CTAS is referred to as a four-stage system as follows:

1. “Critical Look”—A short, several-second assessment according to the ABCD scheme, aimed at quick recognition of patients in priorities 1 and 2
2. Assessment for infectious diseases—Aimed at the rapid isolation of potentially dangerous patients or their decontamination
3. Identify the patient’s main or major complaints, collect objective data (including, for example, vital signs, injury assessment, bleeding severity), use a list of “modifiers”. They are grouped into 17 categories, there are about 177 of them in total, and some of them are additionally graded, so that after confirming a given modifier, the priority can be read from the table
4. The prioritization of the CTAS

An additional advantage is the inclusion of a separate section on children in CTAS.

III. ESI (Emergency Severity Index)

This system was developed in the USA in the 1990s. Unlike the others discussed here, only the first two priorities have the following specific time periods for medical evaluation:

- Immediately for priority 1
- Up to 10 min for priority [25]

It is based on four decision points.

1. Point A—The nurse must assess whether the patient requires life-saving procedures. If so, give priority 1.
2. Point B—Features of a high-risk state for the patient are looked for, the presence of severe pain or disturbances of consciousness—if the result is positive, priority 2 is given.
3. Point C—it is necessary to assess what will be the “consumption” of resources while supplying the patient to the ED. This is a feature that distinguishes the ESI system from others. Patients requiring one or none of the resources receive Priority 4 and 5, respectively. Patients requiring two or more resources go to the next point.
4. Point D—vital parameters are assessed: saturation O_2_, pulse rate, respiratory rate, and temperature in children, based on the table, it is determined whether the patient “promotes” to the priority 2 group or receives priority 3. This point is a kind of “Fuse”, enabling reconsideration of the prioritization of 2 patients [24].

IV. MTS (Manchester Triage System)

It is a system developed in Great Britain, also in the 1990s. Priority is given to specific times for the first contact with a doctor as follows:

- Priority 1 (red)—immediate aid,
- Priority 2 (orange)—up to 10 min,
- Priority 3 (yellow)—up to 60 min,
- Priority 4 (green)—up to 120 min.
- Priority 5 (blue)—up to 240 min [22].

This system is similar to the ATS, which together with the CTAS, forms them into a group of systems based on “discriminators”/“modifiers”, i.e., the confirmation or exclusion of several symptoms or clinical conditions.

What distinguishes it is the presentation of the discriminators in the form of a decision-making algorithm. The nurse initially identifies the patient’s main problem and assigns it to one of 52 cards that reflect the most common reasons for a patient’s presentation to the ED. Then, following the diagram illustrated on the card, individual discriminators should be excluded, grouped, and ranked according to priorities from 1 to 5, until confirmation of the presence of one of the listed ones and the assigned priority can be read.

The scheme forces the focus to first be on proving that the patient is in priority 1, which reduces the triage time in the group of patients urgently in need of help. Vital signs are necessarily measured only if a discriminator is encountered that determines them.

Based on the methodology, the systems can be divided into two groups: based on discriminators, to the confirmation or exclusion of several symptoms or clinical conditions (ATS, CTAS, and MTS), and based on a decision diagram (ESI).

A natural common feature is the desire to ensure that the segregation of patients in Priorities 1 and 2 does not stop the activities in the case of a patient who needs urgent help; that is, it should be conducted as soon as possible.

Therefore, an ideal system of segregation can only be indicated using the criteria from the previous section as shown in Table 1.

## 4. Triage System Comparisons

Considering a variety of systems of triage and their diverse distribution among EDs globally, some attempts have been made to compare them. Such comparisons are difficult for several reasons. These systems differ in terms of the quality and quantity of collected data and endpoints of studies (usually various events during hospitalization). The authors of the published papers compared selected aspects of the systems or simply described their characteristic features. This makes the conclusive analysis of such papers difficult. However, current research analyses focus on the support offered by the use of machine-learning methods rather than a direct comparison of triage systems [32].

The most common systems worldwide are the MTS, CTAS, ESI, and ATS. They are well studied, validated and characterized by high inter-rater reliability—good and very good for ESI and CTAS (kappa 0.7–0.95) and moderated in the case of MTS and ATS (kappa 0.3–0.6) [2,18,33]. An interesting observation arose after the analysis of articles that specifically considered ESI systems. Analyses of different results show that ESI systems seem to be more accurate in a group of children and elderly people than MTS [24,34]. However, some studies indicated that the risk of under-triage increases in the group of patients over 65 years [4,31,35], while Saberian et al. suggested, based on the frailty index, the need to lower this limit to 50 years [36].

Some studies have compared widely accepted triage systems with local nonformalized systems. A good example is the comparison made between ESI with the Taiwan Triage System (TTS), which has shown that ESI predicts better TTS usage of ED resources as well as time spent by patients in the ED and the severity of their conditions [37]. Contrastingly, Zakeri et al. [38] compared the ESI and MTS in a group of trauma patients, showing that the use of the ESI system may generate over triage in a group of patients in priority 3.

Another study performed in a single Dutch ED compared the ESI, MTS, and the Informally Structured System (ISS) [39]. The authors reported a lack of significant differences in the prediction of increased consumption of ED resources, percentage of hospitalizations, and length of hospitalization among patients with the two highest priorities (1/Red and 2/Orange, respectively). The only significant difference in specificity between ESI and MTS was found only in priority 4/Green (postponed service). It was also found that with MTS, the lowest priority (code Blue) was given significantly less frequently than with ESI (code 5). Moreover, the authors of this study noticed the lower sensitivity of ESI and MTS than for earlier publications [28]. This may be because in previous studies, only selected groups of patients, such as critically ill participants, were analyzed [40]. Under triage of patients from the two highest categories described in this study may be a consequence of the methods of assessing the results of triage by experts who know the further course of hospitalization of the participants.

One of the most recent comparison among the three commonly used systems (MTS, ESI, and CTAS) published in 2020 revealed similar sensitivity and specificity for each of the systems [41]. Additionally, structured algorithms of triage were shown to define very precisely and concordantly the highest (1 and 2) and the lowest (5) priorities, while assigning priorities to the intermediate categories (3 and 4) was less precise. Consequently, the most critically ill participants received assistance immediately and effectively, but care for patients assigned to intermediate categories was postponed, while patients still needed urgent treatment in these groups. This is of special concern, as intermediate categories are usually the most abundant in EDs [2].

Regarding the pediatric population, unsatisfactory agreement rates were observed as follows: poor for ATS (0.25), moderate for CTAS (0.571), good for ESI (0.81), and MTS (0.755) [42].

However, in some articles, the highest reliability was found for the ESI and the pediatric version of the CTAS (good inter-rater reliability, with a kappa of 0.8–0.9). The ESI also most accurately predicts the need for hospitalization of a pediatric patient (sensitivity 52%; specificity 81%; AUC 0.78) [34]. Regardless of the reliability of the triage, the ESI is considered a useful tool for handling children in the ED [43].

## 5. Future of Triage

The number of patients seeking help in EDs worldwide is frequently higher than the potential of the EDs themselves. Therefore, a method for improving patient flow is required. Overcrowding of the ED leads to the worsening of the patients’ condition, increase in in-hospital mortality, elongation of stay in hospital, and higher cost [44,45,46]. Immediate knowledge that the patient who is just being admitted to the ED requires a further hospital stay would help to allocate resources in a more optimal way and ensure a more comfortable environment for the patient.

Determining whether ED patients require hospitalization is crucial from both clinical and economic perspectives. To solve this issue, predictive scales based on certain clinical and demographic variables (e.g., age, sex, vital signs, triage priority, etc.) were created [1,47,48,49]. However, attempts have been made to create computational models to facilitate and accelerate the triage process. The task of such a model would be the instant calculation of the probability of patients’ hospitalization [44,46,50]. A perfect situation would be to receive information about the possible or probable hospitalization of patients during triage performed immediately after admission to the ED.

To date, there have been a number of publications with data on the application of machine learning technologies to support segregation processes at different stages. The use of a machine learning algorithm as a predictive model effective in pre-hospital detection of post-traumatic intracerebral hemorrhage (AUC 0.78) based on report data alone is described [13]. In addition, the use of AI-based methods in decision support in the ED is being widely explored. The results of experiments on the ability of machine learning algorithms to predict death in the ED, the need for hospitalization, or ICU admission are promising [51,52,53].

According to some analyses [44,46], the best algorithms for modelling nonlinear relations between variables in the prediction of the need for hospitalization are XGBoosting and deep neural network (DNN). They are fast (taking less than 10 s) [45] and can precisely analyze available data to predict the probability of hospitalization. However, recent reports suggest that DNNs using textual data contained in the available medical documentation of patients can improve the quality of medical triage [19]. Additionally, some studies suggest that the use of modern technological solutions does not have to be difficult or unavailable with the current level of the computerization of medical records but allows for better results, especially in the case of ESI priority [54,55,56].

Machine learning models can also accurately predict serious medical events with vital signs and main symptoms being the most important predictive factors [50,57]. Computational models can also predict the risk of death during hospitalization better than triage systems alone [47,50]. According to various researchers, good discriminatory results for sudden cardiac arrest (SCA) in the ED, obtained solely from triage data, were obtained using Random Forest (AUC 0.931) [53], Logistic Regression (AUC 0.925) [15], and Ada Boosting and Light Gradient Boosting machine (AUC 0.997) [58].

An additional advantage of such models is a more exact triage, with a lower percentage of under-triaged patients appearing in priorities 1–3 and over-triage in priorities 3–5 [50].

However, regardless of the type of AI-based algorithm being tested, the most common gold standard was the assessment by an experienced medical professional [8,59,60]. It is worth noting that several papers provide evidence that, although the sensitivity and specificity of the diagnostic assessment is acceptable in predicting hospitalization, the accuracy for mental illness is the lowest [60]. Recently, articles have been published describing the use of AI-based language models to assist in medical segregation in the emergency department. It was found that both the Generative Pre-trained Transformer GPT-4 and Gemini can accurately triage critical and urgent ESI group 1 and 2 patients, suggesting that both models can help accurately segregate these patients in the ED [61].

An important but rarely discussed problem in emergency departments is patients who leave without being seen before medical segregation. It has been shown that AI can be used to predict which patients leave the ED without being seen, which can enable trajectories of their stay to be altered and can influence individual decisions of ED staff [62].

A separate issue being investigated in the survey among emergency physicians is their attitude towards the AI tool. Researchers from Turkey showed that there is a strong conviction among ED staff about the benefits of using AI support to assess a patient during triage; however, concerns about the related ethical aspects of such an intervention are also present among them [63]. Some of the misunderstandings about the idea of machine learning are addressed by the publications discussing the topic in more depth in the emergency medicine group [64].

Summarizing the comparison between computational models and traditional triage systems, the authors state that artificial intelligence is more effective in predicting the main final points (hospitalization, admission to ICU, and death) [15,50,51,52,53,57,58]. Moreover, they can improve the quality of care in the ED and reduce the burden on healthcare systems [32].

## 6. Summary

Nowadays, it is difficult to indicate which of the segregation systems available worldwide is the best. To approximate a remedy to this issue, the authors reviewed several publications, comparing triage systems to select the most optimal system for use in the ED [5,65,66].

The most popular existing triage systems can be divided into diagrams with disease symptoms, containing discriminating criteria (ATS, MTS, CTAS), and using one decision algorithm for all patients (ESI).

The training process of each algorithm appears to be equally important in each triage system. In the available publications, little data are used to compare the training schemes for different segregation systems. Many studies in the methodology state that employees performed the triage after several hours of training in each algorithm. However, in practice, professionals trained over a longer period do seem to use these systems more efficiently.

## 7. Limitations

A significant limitation of this study is the lack of widespread clinical practice related to the use of machine learning methods, including the disproportion in the number of available reports on the effectiveness of traditional triage systems compared to artificial intelligence techniques. Another important limitation is that in the analyzed publications, the evaluation criteria for the triage systems were not the same or comparable. The incompatibility of the methodologies for describing the segregation schemes in the publications resulted in an imperfect and slightly informative approach to comparing these systems.

## 8. Conclusions

According to a review of the literature, this study concludes that triage systems existing in the world increase the safety of patients waiting for assistance in the ED. It would seem obvious that any system for segregating patients admitted to the ED is better than none; however, numerous studies have shown the superiority of five-step systems over the three-step systems, mainly in terms of reliability. After nearly 30 years of development, the maximum efficiency of the above-mentioned systems seems to have been achieved. Based on the authors’ experience, medical segregation, even when handled professionally and using a certified system, does not guarantee 100% safety in regard to unexpected medical events.

Another issue is the process of evaluating the used system, which is inextricably linked to maintaining the proper quality of the process. To compile the learning time required to effectively use each of these systems would be advised as no in-depth studies have been conducted on this topic.

Each of these systems lacks a parameter/criterion that has not yet been considered. To the question of what this additional value could be, the answer may be the results of work on machine learning systems, which very accurately predict the risk of hospitalization, both in the ward and in the ICU, in a much faster time than a human would do. Based on calculations obtained by the AI method, one could add in the case of diagram-based systems (MTS, CTAS, ATS), a specific differentiation criterion, and in the case of algorithmic systems (ESI), an additional parameter at decision point C.

In summary, the authors in this study did not find an answer to the question of which triage methodic is the best. Currently, machine learning seems to provide more opportunities for the unconventional analysis of data available during medical triage. Possibly, an ideal segregation system, or rather a decision-making process including medical segregation, will be a combination of the experience and intuitions of trained medical personnel and modern technology, including artificial intelligence methods based on the analysis of the large amount of data.

## Figures and Tables

**Table 1:** Table subjectively comparing the most popular medical triage systems with authors’ comments (+ good), (++ very good), (? not specified).

	ATS	CTAS	ESI	MTS
Validity	+/? Little research [2]	+/? Good [2]	+/? [2] Different results analyses in research, though seems more accurate in a group of children and elderly people than MTS [19,21,22]	+/? [2] Contradictory data depending on the study. Probably depending on the age of the patient and distance hospital from place uprising triage system
Reliability Kappa value	0.25 to 0.56, but decreases in the group of patients with mental disorders [2]	Adults 0.68–0.89 Children 0.51–0.72 [2]	Adults 0.46–0.91 Children 0.82 [2]	Adults 0.31–0.62 [2]
Simplicity (subjective evaluation)	++	+	+(+) Seems to depend on the presence of clear guidelines (diagnostic and treatment standards) in the hospital	++
Short time of performance	Time depends on patient priority, the higher the priority the shorter the time	Time depends on patient priority, the higher the priority the shorter the time	Time depends on patient priority, the higher the priority the shorter the time	Time depends on patient priority, the higher the priority the shorter the time
Formalized structure	+	+	+	+

## Data Availability

The original contributions presented in the study are included in the article, further inquiries can be directed to the corresponding authors.
